# Accessibility and usage patterns of wearable devices among Chinese adults: the Huawei Blood Pressure Health Study

**DOI:** 10.1093/ehjdh/ztaf088

**Published:** 2025-08-05

**Authors:** Ying Wang, Shan-Shan Zhou, Yu-Qi Liu, Dan-Dan Li, Shun-Ying Hu, Xi Wang, Li Yi, Ya-Ni Yu, Yun-Dai Chen

**Affiliations:** Department of Cardiology, The Sixth Medical Centre, Chinese PLA General Hospital, No. 6 Fucheng Road, Haidian District, Beijing 100048, China; Department of Cardiology, The Sixth Medical Centre, Chinese PLA General Hospital, No. 6 Fucheng Road, Haidian District, Beijing 100048, China; Department of Cardiology, The Sixth Medical Centre, Chinese PLA General Hospital, No. 6 Fucheng Road, Haidian District, Beijing 100048, China; Department of Cardiology, The Sixth Medical Centre, Chinese PLA General Hospital, No. 6 Fucheng Road, Haidian District, Beijing 100048, China; Department of Cardiology, The Sixth Medical Centre, Chinese PLA General Hospital, No. 6 Fucheng Road, Haidian District, Beijing 100048, China; Department of Cardiology, The Sixth Medical Centre, Chinese PLA General Hospital, No. 6 Fucheng Road, Haidian District, Beijing 100048, China; Department of Cardiology, The First Medical Centre, Chinese PLA General Hospital, No. 28 Fuxing Road, Haidian District, Beijing 100039, China; Department of Cardiology, The Sixth Medical Centre, Chinese PLA General Hospital, No. 6 Fucheng Road, Haidian District, Beijing 100048, China; Department of Cardiology, The Sixth Medical Centre, Chinese PLA General Hospital, No. 6 Fucheng Road, Haidian District, Beijing 100048, China

**Keywords:** blood pressure, Wearable devices, Hypertension, Real-world study, Accessibility, Digital divide

## Abstract

**Aims:**

This study aims to investigate the ownership of wearable health devices across different demographic groups and usage patterns among Chinese adults.

**Methods and results:**

This was a cross-sectional study, with all data originating from the Huawei Blood Pressure Health Study, a real-world study aimed at exploring blood pressure management through wearable devices in China. Data were remotely collected using mobile phones and Huawei Watch D from 23 February 2022 to 31 March 2024. The system utilized artificial intelligence algorithms to assess participants’ risk of hypertension and provided risk alarm feedback via mobile phones and watches. A total of 75 918 participants from 31 provinces were included, with an average age of 47 years. Most of the participants were concentrated in the economically developed South China and East China regions. Among the participants, 73.8% used the Watch D for blood pressure monitoring, and 10.5% received risk alerts. The rate of blood pressure monitoring on the day they received the alert was 78%. However, the rate significantly decreased between 6 months and 1 year (Mann–Kendall test, *Z* = −2.85, *P* < 0.05). For hypertensive patients, the blood pressure monitoring rate was 84% on the day they joined the study and decreased over time (Mann–Kendall test, *Z* = −3.09, *P* < 0.05). However, it remained above 50% within 6 months.

**Conclusion:**

This study provides evidence of the digital health divide in the utilization of wearable devices among the Chinese population. Additionally, it proposes a potentially follow-up interval for employing wearable devices for maintaining compliance with blood pressure monitoring.

**Study registration:**

URL: https://www.chictr.org.cn/

**Unique identifier for Huawei-BPHS:**

ChiCTR2200057354

## Graphical Abstract

**Graphical Abstract:**
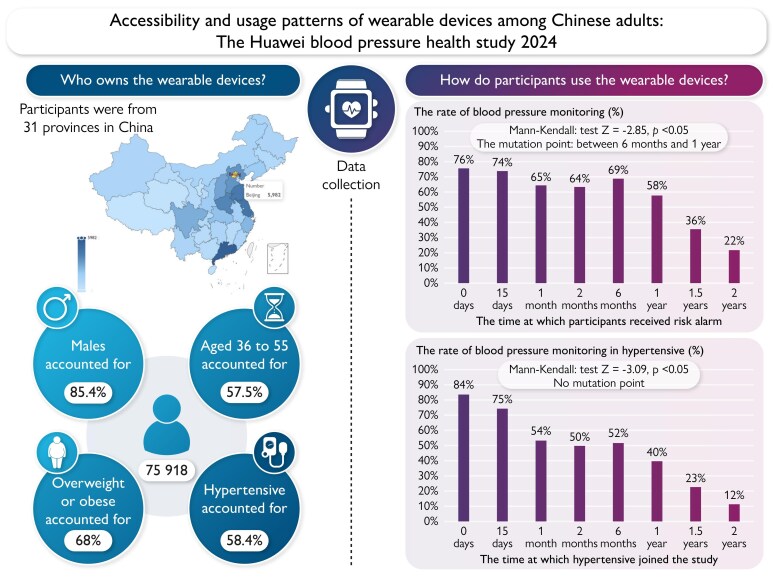

## Introduction

Hypertension is one of the major risk factors for cardiovascular diseases.^1^ Despite the advent of numerous safe and effective antihypertensive medications in recent decades, the global control rate for hypertension remains suboptimal, particularly within developing nations.^2,3^ The cornerstone of effective blood pressure management hinges on prompt diagnosis, which is then followed by tailored therapeutic interventions and lifestyle modifications. Moreover, continuous monitoring of blood pressure and adherence to prescribed treatment regimens are imperative. Current clinical guidelines recommend for the integration of out-of-office blood pressure measurement techniques, including 24-h Ambulatory Blood Pressure Monitoring (ABPM) and Home Blood Pressure Monitoring (HBPM), to enhance the precision and personalization of hypertension care.^4–7^ These approaches, however, encounter practical constraints such as restricted frequency of measurements, limited accessibility, and user discomfort. In the digital age, direct-to-consumer (D2C) wearable technologies have addressed several shortcomings associated with conventional methods. By facilitating more frequent and less obtrusive blood pressure readings across diverse environments and daily routines, these innovative devices hold substantial promise for enhancing long-term adherence to treatment plans and improving outcomes in hypertension management.^8^

To effectively utilize wearable devices in blood pressure management, two important issues need to be considered: accessibility and compliance.^9^ The World Health Organization (WHO) defines healthcare service accessibility as emphasizing fairness and availability of healthcare services for all.^10^ However, the existence of digital divide and digital health literacy levels may limit the use of D2C wearable devices among certain groups with lower incomes and education levels, and respondents living in rural areas.^8,11,12^ Another significant issue is indeterminate compliance among users. For instance, the average duration of app engagement was only 4.1 days within the 6-month study period of the MyHeart Counts study.^13^ This finding parallels results observed in both the Asthma Health and mPower studies.^9,14,15^ In the Apple-Heart study, only 43% participants completed an end-of-study survey.^16^ On the other hand, a longer-term study with an observation period exceeding 31 months found that 75% of users re-engaged with the device after a period of idleness following its purchase, and their usage patterns resembled those during their initial use of the app activity tracking applications.^17^ Nonetheless, long-term user engagement patterns in real-world blood pressure monitoring are not yet well understood.

Therefore, this study utilizes 2 years of monitoring data from the Huawei-BPHS to describe the accessibility characteristics of populations with blood pressure monitoring-enabled wearable devices, as well as their usage patterns among the general population. It may provide evidence of a digital health divide within the Chinese population concerning the use of wearable devices for blood pressure monitoring. This serves as a foundation to drive changes in healthy behaviours and optimize management strategies for hypertension.

## Methods

### Study design and participants

This was a cross-sectional study utilizing data from the Huawei-BPHS, a nationwide, wearable devices-based study to manage blood pressure in large population across China. This study was developed by the China International Exchange and Promotive Association for Medical and Healthcare, with support from Huawei Technologies Co., Ltd., and is being implemented by the Chinese People’s Liberation Army General Hospital, Beijing, China. Data were remotely collected from 23 February 2022 to 31 March 2024. All participants voluntarily signed the electronic research declarations, privacy agreements, and informed consent forms remotely though ‘Huawei Innovation Research App’. For this study, the inclusion criteria for online participant recruitment were as follows: age between 18 and 80 years; wrist circumference between 130 and 200 mm; Chinese nationality and residency within China; proficiency in Chinese writing, reading, and communication; ability to use the Huawei Watch D and the ‘Huawei-BPHS’ app without difficulty. The exclusion criteria were as follows: presence of wrist injuries or dark tattoos; pregnancy; severe arrhythmia (e.g. atrial fibrillation, frequent premature beats, severe conduction blocks); severe shock or the use of cardiopulmonary bypass; presence of upper limb infections or bleeding tendencies. The study was approved by the General Hospital of the Chinese People’s Liberation Army Ethics Committee (S20221-567), and conducted in accordance with the principles of the World Medical Association Declaration of Helsinki. The study was registered at https://www.chictr.org.cn/(ChiCTR2200057354). The study procedures are placed in Supplementary material online, material  *S1*.

### Variable collection and definition

This study employed mobile phones and Huawei Watch D for data collection. The Watch D has achieved Class II medical device certification (No.: 20212071428) by the National Medical Products Administration.^18^ The accuracy of blood pressure measurements obtained using the Huawei Watch D conforms to the ANSI/AAMI/ISO 81060-2:2018 validation standards in both resting and active states of participants, and this has been confirmed through multiple-centre clinical trials.^19–21^ Blood pressure parameters (including systolic blood pressure, diastolic blood pressure, circadian rhythm, number of risk alerts, daily proportion of abnormal blood pressure, hourly proportion of abnormal blood pressure, time of highest abnormal blood pressure, etc.), sleep parameters (including earliest bedtime, latest bedtime, light sleep duration, deep sleep duration, dream duration, total sleep duration, wakefulness frequency, daytime sleep duration, etc.), exercise parameters (including physical activity duration, energy expenditure, exercise distance, steps, activity types, etc.), stress parameters, and heart rates were objectively and continuously monitored by the Huawei Watch D, while demographic information and disease history were obtained through electronic questionnaires on the ‘Huawei-BPHS’ app. Weight (kg) and height (m) were reported by participants and used to calculate body mass index (BMI). All the data were uploaded to the Blood Pressure Health Study Cloud.

The American Sleep Foundation recommended that adults sleep for 7–9 h per night.^22^ In this study, 7–9 h was used as the reference value, and ‘<5 h and 5–7 h’ was defined as short sleep duration, and ‘>9 h’ was defined as the long sleep duration. Metabolic equivalents (METs) refer to a unit of measure that is used to express the energy cost of physical activities, where one MET is defined as the rate of energy expenditure at rest. The calculation formula is as follows: MET = energy expenditure/body weight (kg)/physical activity duration (h). This concept provides a standardized way to compare the intensity of various activities. MET was calculated as the above formula and stratified into at rest (1 MET), light activity (2 METs), moderate activity (3–6 METs), and vigorous activity (>6 METs).^18^ Participants were stratified into infrequent participation (1–10 times), regular participation (10–43 times), active participation (43–142 times), and very active participation (≥142 times) according to the interquartile range of blood pressure monitoring frequencies.

### Data access and cleaning

Data collection adheres to principles of user consent, data minimization, and legitimacy verification. Data storage is encrypted at both hardware and user levels on the cloud side, while data transmission is secured via hypertext transfer protocol secure (HTTPS). Data sharing is contingent on user authorization, and personalized content recommendations can be disabled. The information security measures have passed the national Information Security Protection Level Three certification and comply with global privacy laws, including the Personal Information Protection Law of the People’s Republic of China and the General Data Protection Regulation (GDPR) in Europe.

The dataset utilized in this research was procured through a collaboration with Huawei Technologies Co. Ltd. and the data for this study were securely stored in an offline MySQL database. Access to this data was strictly controlled, with only duly authorized researchers granted permission to utilize it. Data pre-processing encompassed several steps to ensure the integrity and accuracy. Individuals who had not disclosed their age or whose reported age falls outside the range of 18–80 years were excluded from the study. Similarly, entries with unspecified gender were omitted. The BMI values were sorted in ascending order. From this sorted list, the BMI value corresponding to the first percentile (16.8889) and the 99th percentile (36.9272) were identified. Any individual with a BMI of <16.8889 or >36.9272 was considered an outlier and further excluded from the dataset.

### Statistics

For continuous variables, descriptive statistics will be presented as mean scores and standard deviations (SDs) if the data follow a normal distribution, or as medians with interquartile ranges for skewed distributions. Binary and categorical variables will be summarized using frequency distributions and corresponding percentages. The interquartile range of blood pressure monitoring frequencies was calculated for 56 005 participants who had their blood pressure monitored at least once upon joining the study. The adherence to blood pressure monitoring was calculated in those received risk alarm and hypertensive patients. The Mann–Kendall Trend Test, a non-parametric test, was employed to assess whether the rate of blood pressure monitoring has a monotonic upward or downward trend over time.^23^ All statistical tests were two-sided, with a significance level set at *P* < 0.05. Statistical analysis was performed using Python 3.12.5.

## Results

### Characteristics of study population

**Figure 1:**
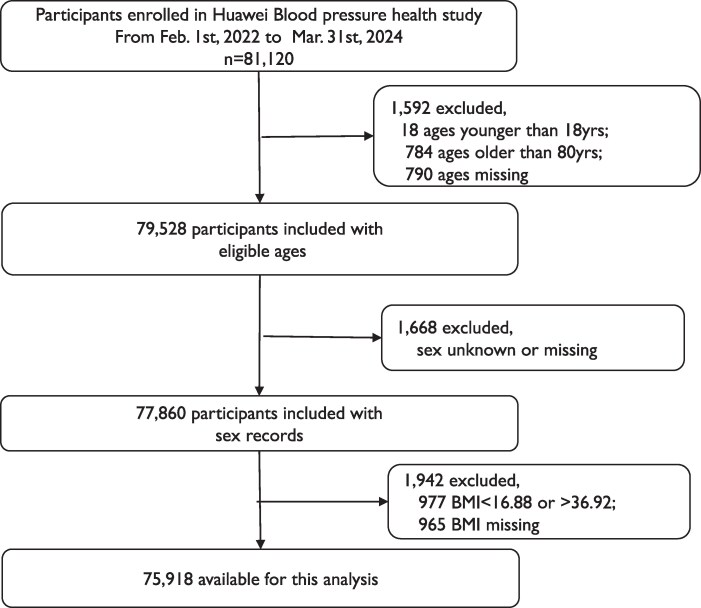
The flow chart of study population. BMI, body mass index.

**Figure 2:**
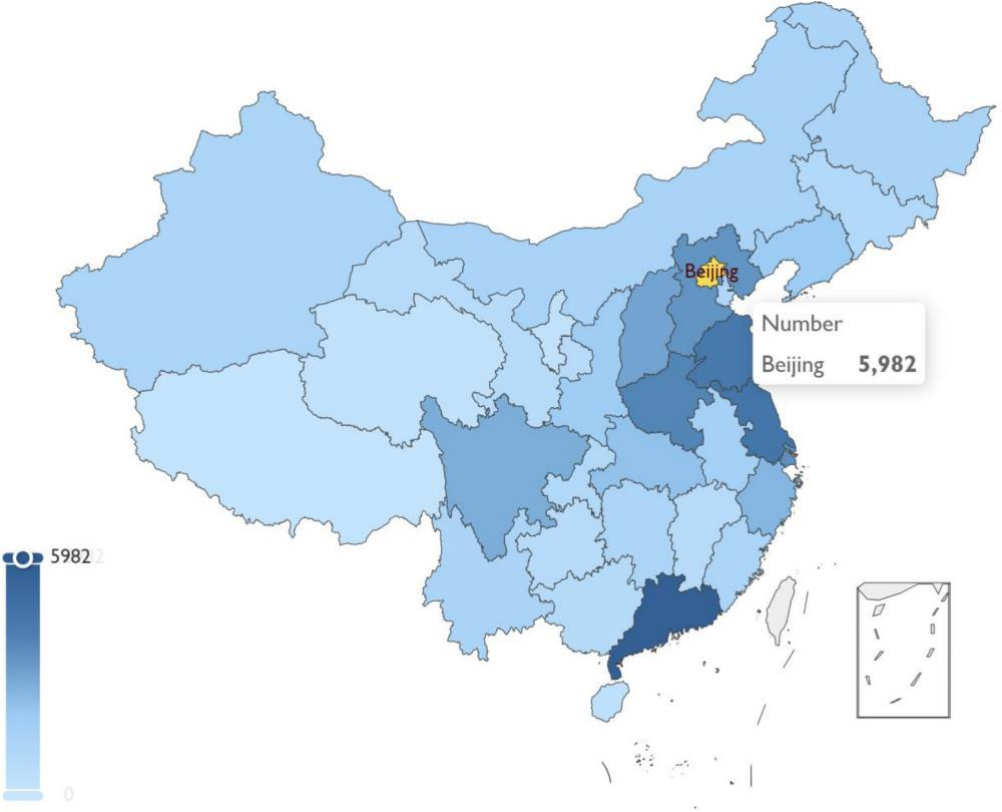
Geographical distribution of participants within the Huawei Blood Pressure Health Study.

A total of 75 918 participants from 31 provinces were included in the study (*Figure 1*), 23.7% (17 999) participants completed the electronic questionnaire for hypertension. The demographic characteristics of these participants exhibited significant regional features (*Figure 2*). Beijing had the highest number of participants, with 5982 cases. Most participants were primarily concentrated in the economically developed South China and East China regions. The characteristics of the participants are detailed in *Table 1*. Overall, males comprised 85.4% (63 858) of the sample. The average age of the participants was 47 years, with 68% (51 622) being classified as overweight or obese. Additionally, 58.4% (10 504) of the participants suffered from hypertension. Among the participants, 73.8% (56 005) monitored their blood pressure, 70.0% (52 379) monitored their sleep, and only 19.9% (15 094) participants had energy expenditure, body weight, and physical activity duration record, which were used to classify the physical activity intensity. Among those who underwent sleep and active physical activity monitoring, 99.7% of users monitored their blood pressure.

**Table 1:** The characteristics of study population enrolled in the Huawei Blood Pressure Health Study

	Male	Female	Total
	*n* = 63 858	*n* = 11 060	*n* = 75 918
Age (years)	46.7 ± 11.7	50.1 ± 14.4	47.2 ± 12.2
Age (*n*, %)			
18–35	11 098 (17.1)	2100 (19.0)	13 198 (17.4)
36–45	19 886 (30.7)	1980 (17.9)	21 866 (28.8)
46–55	19 034 (29.3)	2765 (25.0)	21 799 (28.7)
56–65	10 604 (16.3)	2407 (21.8)	13 011 (17.1)
66–75	3723 (5.7)	1547 (14.0)	5270 (6.9)
＞75	513 (0.8)	261 (2.4)	774 (1.0)
BMI (*n*, %)			
<18.5	421 (0.6)	356 (3.2)	777 (1.0)
18.5–24	17 926 (27.6)	5593 (50.6)	23 519 (31.0)
24–28	30 123 (46.4)	3758 (34.0)	33 881 (44.6)
>28	16 388 (25.3)	1353 (12.2)	17 741 (23.4)
Hypertension^a^ (*n*, %)			
Yes	9435 (59.8)	1069 (48.2)	10 504 (58.4)
No	3656 (23.2)	739 (33.3)	4395 (24.4)
Uncertain	2689 (17.0)	411 (18.5)	3100 (17.2)
Sleeping duration^b^			
<5 h	5409 (11.9)	474 (6.8)	5883 (11.2)
5–7	30 387 (66.9)	4176 (59.9)	34 563 (66.0)
7–9	9109 (20.1)	2172 (31.1)	11 281 (21.5)
>9	499 (1.1)	153 (2.2)	652 (1.2)
Physical activity^c^			
At rest	557 (4.0)	126 (11.1)	683 (4.5)
Light activity	1596 (11.4)	265 (23.3)	1861 (12.3)
Moderate activity	10 123 (72.5)	673 (59.2)	10 796 (71.5)
Vigorous	1681 (12.0)	73 (6.4)	1754 (11.6)

BMI, body mass index.

^a^A total of 17 999 (23.7%) participants completed the electronic questionnaire for hypertension.

^b^52 379 (70.0%) participants monitored sleeping duration.

^c^15 094 (19.9%) participants had energy expenditure, body weight and physical activity duration, which were used to calculated metabolic equivalents (MET). Physical activities were stratified into at rest, light activity, moderate activity, and vigorous activity according to the level of metabolic equivalents (MET).

### Characteristics of participants in different levels of blood pressure monitoring

**Figure 3:**
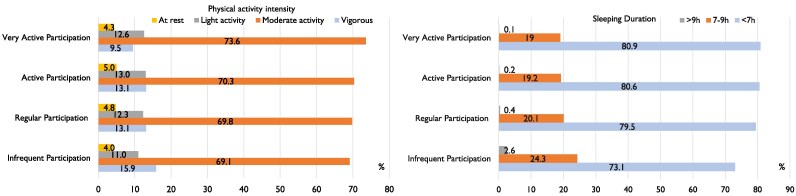
The characteristics of physical activity intensity and sleep duration according to different levels of blood pressure monitoring (*n* = 56 005).

The frequency of blood pressure monitoring increased with the advancing age of participants. In the very active participation group, individuals aged 45–65 years accounted for 55.1%, a significantly higher proportion compared with other groups. Additionally, this group had a higher percentage of males, overweight individuals, and those previously diagnosed with hypertension (*Table 2*). As the frequency of blood pressure monitoring among participants increased, the proportion of individuals with shorter sleep durations and those engaging in moderate-intensity physical activities also rose (*Figure 3*).

**Table 2:** The characteristics of participants according to different levels of blood pressure monitoring (*n* = 56 005^a^)

	Infrequent participation^b^	Regular Participation	Active Participation	Very Active Participation
	*n* = 13 321	*n* = 14 603	*n* = 14 066	*n* = 14 015
Sex (*n*, %)				
Male	10 919 (82)	12 540 (85.9)	12 356 (87.9)	12 556 (89.6)
Female	2402 (18)	2063 (14.1)	1710 (14.1)	1459 (10.4)
Age (years)	46.4 ± 12.9	46.1 ± 12.1	47 ± 11.6	49.6 ± 11.1
Age (*n*, %)				
18–35	2777 (20.8)	2774 (19)	2137 (15.2)	1326 (9.5)
36–45	3738 (28.1)	4472 (30.6)	4436 (31.5)	3692 (26.3)
46–55	3489 (26.2)	4113 (28.2)	4288 (30.5)	4790 (34.2)
56–65	2217 (16.6)	2275 (15.6)	2217 (15.8)	2934 (20.9)
66–75	949 (7.1)	851 (5.8)	861 (6.1)	1126 (8)
＞75	151 (1.1)	118 (0.8)	127 (0.9)	147 (1.1)
BMI (*n*, %)				
<18.5	162 (1.2)	169 (1.2)	93 (0.7)	86 (0.6)
18.5–24	4205 (31.6)	4271 (29.2)	4126 (29.3)	4580 (32.7)
24–28	5678 (42.6)	6411 (43.9)	6438 (45.8)	6693 (47.8)
>28	3276 (24.6)	3752 (25.7)	3409 (24.2)	2656 (18.9)
Hypertension (*n*, %)				
Yes	1206 (54.6)	1864 (55.6)	2518 (56.6)	3980 (62.4)
No	676 (30.6)	953 (28.4)	1127 (25.3)	1187 (18.6)
Uncertain	328 (14.8)	536 (16)	804 (18.1)	1215 (19)

BMI, body mass index.

^a^A total of 56 005 (73.8%) participants monitored the blood pressure.

^b^Participants were stratified into infrequent participation (1–10 times), regular participation (10–43 times), active participation (43–142 times), and very active participation (≥142 times) according to the interquartile range of blood pressure monitoring frequencies.

### Compliance with blood pressure monitoring

**Figure 4:**
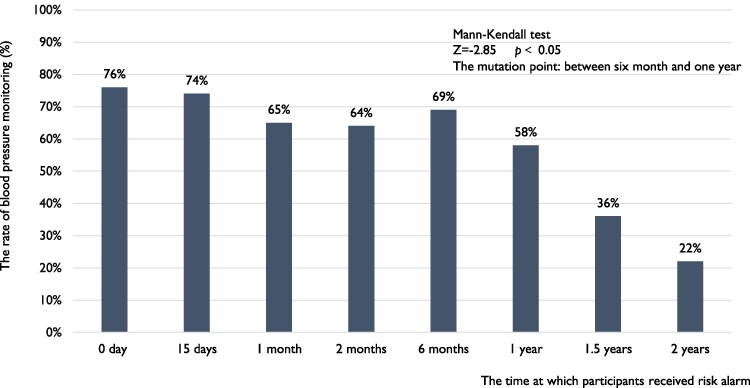
The rate of blood pressure monitoring for participants received risk alerts. ‘The rate of blood pressure measurement’ refers to the percentage of participants who measure their blood pressure at least once; each bar indicates the rate of blood pressure measurement between two adjacent time points. ‘Day 0’ refers to the day from when participants receive a risk alert of high blood pressure.

**Figure 5:**
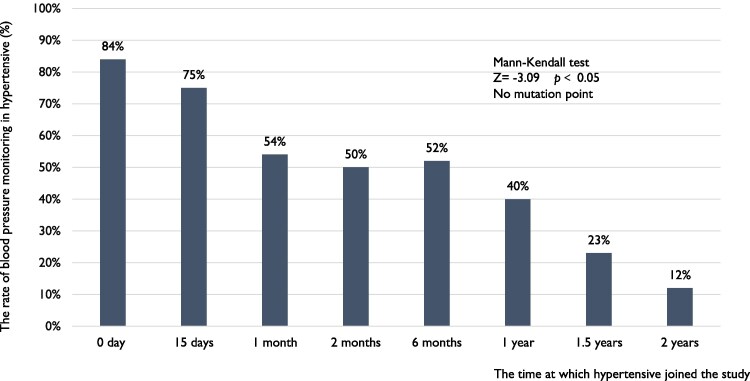
The rate of blood pressure monitoring in hypertensive. ‘The rate of blood pressure measurement’ refers to the percentage of participants who measure their blood pressure at least once; each bar indicates the rate of blood pressure measurement between two adjacent time points. ‘Day 0’ refers to the day from when hypertensive joined the study.

The rate of blood pressure monitoring among individuals who received a hypertension risk alert was 76% on the day they received the alert, and remained at 74% within 2 weeks (*Figure 4*). However, this rate showed declining trend over time (Mann–Kendall test, *Z =* −2.85, *P* < 0.05), significantly dropping between 6 months and 1 year. The mutation point of blood pressure monitoring rate was 9 months (see Supplementary material online, *F*igure  *S1*). For patients with diagnosed hypertension, the rate of blood pressure monitoring on the day they joined the study was 84%, and their compliance with monitoring decreased over time (Mann–Kendall test, *Z =* −3.09, *P* < 0.05, *Figure 5*) and had no mutation point (see Supplementary material online, *F*igure  *S2*). Nevertheless, it remained above 50% within 6 months.

### The relationship between the frequency of blood pressure monitoring and the number of hypertension risk alerts received

**Figure 6:**
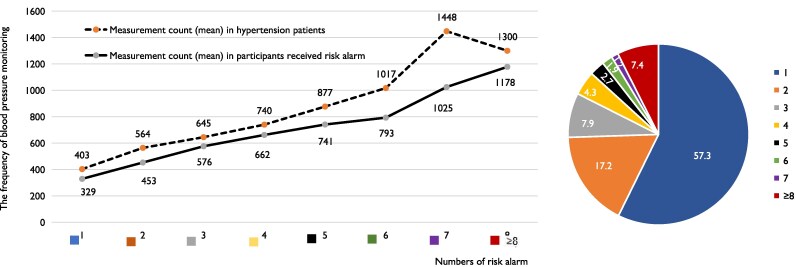
The relationship between the frequency of blood pressure monitoring and numbers of risk alerts received.

In the study population, 10.5% received risk alerts, 20.1% of the hypertensive population received risk alerts. Among those who received risk alerts, 57.3% received one alert (*Figure 6*). The average frequency of monitoring increased with each additional risk alert received, both in the overall participants and specifically among individuals with hypertension. However, a notable decline in average monitoring frequency was observed for hypertensive patients after they received their seventh alert.

## Discussion

This study represents the first and largest real-world investigation into the application of wearable devices in blood pressure management. This cross-sectional study reveals the ownership and usage patterns of wearable devices equipped with blood pressure monitoring capabilities among the general population in China. Our findings provide evidence to support the widespread adoption of this technology in the field of blood pressure management.

This study uncovers the digital health divide among wearable device users in China. After analysing 2 years of continuous monitoring data, it was found that economically developed South China and East China had more owners of the Huawei Watch D. Most of these owners were concentrated in central cities including Beijing, Shanghai, Shenzhen, and Guangzhou. The primary demographic comprised males aged between 35 and 65 years, with a notable prevalence among those who are overweight or obese. Conversely, female users constituted a mere 14.6% of the total user base, indicating a significant gender disparity in adoption patterns. Previous researches have demonstrated that the uptake of digital health management tools is significantly influenced by factors such as gender, age, and socioeconomic standing, with additional pronounced disparities observed across different racial and ethnic groups. A study from Australia indicated that factors such as increasing age, residing in lower socioeconomic areas, male, and lack of higher education might all hinder individuals’ access to digital health services.^11^ On the contrary, men were less likely to seek eHealth care compared with women in the USA^24^ These findings underscore the complex interplay of demographic variables in shaping access to and utilization of digital health technologies, emphasizing the need for tailored interventions to address these inequities. If wearable devices are to be equitably applied in health monitoring, it depends on support from multiple sectors, including network accessibility,^25^ device availability, and public health awareness levels.

The blood pressure monitoring function of wearable devices was not fully utilized. Users who receive risk alerts and patients with hypertension maintain a satisfactory level of adherence to blood pressure monitoring for a period, but the rate of blood pressure monitoring significantly declined over time. By the 2-year mark, less than one-fifth of individuals were still using smartwatches to monitor their blood pressure. Therefore, identifying appropriate follow-up intervals is crucial for timely supervision to maintain or improve the rate of blood pressure monitoring among high-risk populations and those with hypertension. Expert consensus and guidelines recommended that new remote medical technologies can improve blood pressure management, potentially allowing extended intervals between visits.^4,26^ However, currently, there is no data to support this. This study suggests potential follow-up intervals for employing wearable technology in blood pressure monitoring for high-risk and hypertension patients. Our study found that among individuals received risk alerts, the rate of blood pressure monitoring decreases over time from the day they receive the alert. The rate showed a significant declining trend between 6 months and 1 year. For hypertensive patients, adherence to blood pressure monitoring at 6 months significantly drops. It is suggested that follow-ups should be conducted annually for those received risk alerts, and biannually for hypertensive patients, to improve and sustain adherence to blood pressure monitoring protocols.

Sleep quality and regular physical activity are key factors in maintaining stable blood pressure.^27^ Traditional research on sleep and exercise data relied mainly on questionnaires, which were susceptible to recall bias. However, in this study, we used the Watch D device to monitor users’ sleep and activity levels objectively and continuously, which was significant for a deeper understanding of the relationship between sleep, exercise, and blood pressure fluctuations. Despite this, it was noteworthy that only 68% of the target population enabled the sleep monitoring function, and even fewer, at 12%, enabled the exercise monitoring function. This indicates that a large portion of the population has not fully utilized this advanced health tracking tools, potentially missing out on better managing their health. Therefore, raising public awareness of these functions and their potential benefits is particularly important. When examining lifestyle factors alongside monitoring habits, it was observed that as the frequency of blood pressure monitoring increases, there was a corresponding rise in the proportion of individuals with shorter sleep durations and who engaged in moderate-intensity physical activities. This suggests an interaction between health awareness, preventive measures, and lifestyle choices among individuals who were particularly complying with their blood pressure monitoring. It will be analysed in our future research.

There are some limitations to this study. First, the sample of this study primarily consists of users of Huawei devices, whose lifestyle habits, health conditions, and age distribution may differ from those using other brands. This selection bias could influence the generalizability of the research findings. Second, 15% of the users who purchased the device lacked blood pressure data, mainly due to the following reasons: blood pressure values are failure to synchronize data via Bluetooth or the cloud; users who joined the study but did not grant permission to the App for accessing to data; and the local data exceeding retention days without timely upload to the cloud by users. This highlights the need to further optimize the data synchronization process of blood pressure monitoring devices. Third, parameters such as sleep and exercise, which were previously dependent on self-reported data, can now be collected objectively and in real-time by wearable devices in this study, significantly reducing recall bias. However, the collection of medical histories continued to depend on self-reported electronic questionnaires. Only 23.7% of participants completed the electronic questionnaire for hypertension, which may introduce recall bias. Fourth, this study revealed that patients with hypertension constituted a larger proportion of owning the watch and conducted blood pressure monitoring more frequently. However, given the cross-sectional nature of this study, we cannot infer causal relationships between hypertension and being a super tracker.

## Conclusions

This study reveals the digital health divide and identifies that the functionalities of wearable health devices are underutilized among Chinese populations. Based on the patterns observed in participants’ use of wearable devices, we propose potential follow-up intervals for blood pressure management in high-risk hypertension groups and patients with hypertension. Annual follow-ups might be recommended for high-risk groups, while biannual follow-ups might be advised for those with hypertension to ensure timely monitoring and maintain adherence to blood pressure tracking.

## Supplementary Material

ztaf088_Supplementary_Data

## Data Availability

The data underlying this article were provided by Huawei Technologies Co., Ltd. by permission. Data will be shared on request to the corresponding author with permission of Huawei Technologies Co., Ltd.

## Lead author biography

Dr Yun-dai Chen currently serves as the Professor and Director of Department of Cardiology, Chinese PLA General Hospital. She serves as the Vice President of the Chinese Medical Doctor Association’s Cardiology Branch, the editor-in-chief of the *Journal of Geriatric Cardiology*, the associate editor of the *Journal of the American College of Cardiology: Asia*. She is the leader of the Huawei Blood Pressure Health Study, her academic efforts concentrate on exploring a new mode for the management of chronic non-infectious diseases based on AI technology.
