# Supplementary material for: Accessibility and usage patterns of wearable devices among Chinese adults: the Huawei Blood Pressure Health Study
Source: Eur Heart J Digit Health. 2025 Aug 5;6(6):1264–72. doi: 10.1093/ehjdh/ztaf088 (PMC12629657; doi:10.1093/ehjdh/ztaf088)

**Supplemental material**

1. **Study procedures**

The study was structured into three distinct phases. *Participant Recruitment:* Participants were required to download the ‘Blood Pressure Health Study’ application on their smartphone and follow the provided guidelines to pair and connect the Watch D device. Upon successful connection, participants voluntarily signed the research declaration, privacy agreement, and informed consent forms. Eligible volunteers, determined by predefined inclusion and exclusion criteria, were enrolled in this study. *Data Collection:* Participants were instructed to self-report personal information, including demographic characteristics and medical history. Blood pressure monitoring commences once participants wore the watch and completed the calibration of the blood pressure measurement according to the operational instructions. Watch D continuously and automatically monitors blood pressure, heart rate, blood oxygen levels, sleep patterns, physical activity, and other physiological parameters with participant consent. Participants were encouraged to monitor their blood pressure for a duration exceeding one week. *Hypertension Risk Assessment:* The risk of hypertension was assessed using an artificial intelligence algorithm within the “Blood Pressure Health Study app”1. This approach incorporates a wide array of variables, including blood pressure values obtained through the oscillometric method, comprehensive pulse pressure waveform characteristics across both frequency and temporal dimensions, as well as crucial demographic information like age and gender (as the diagram shown). Within 48 hours after completing the blood pressure calibration task, a reminder will be automatically sent every two hours by default if the algorithm detects any features in the PPG waveform suggesting a “suspected elevated blood pressure”. Personalized lifestyle recommendations also sent to those participants to encourage lifestyle modifications aimed at reducing hypertension risk.


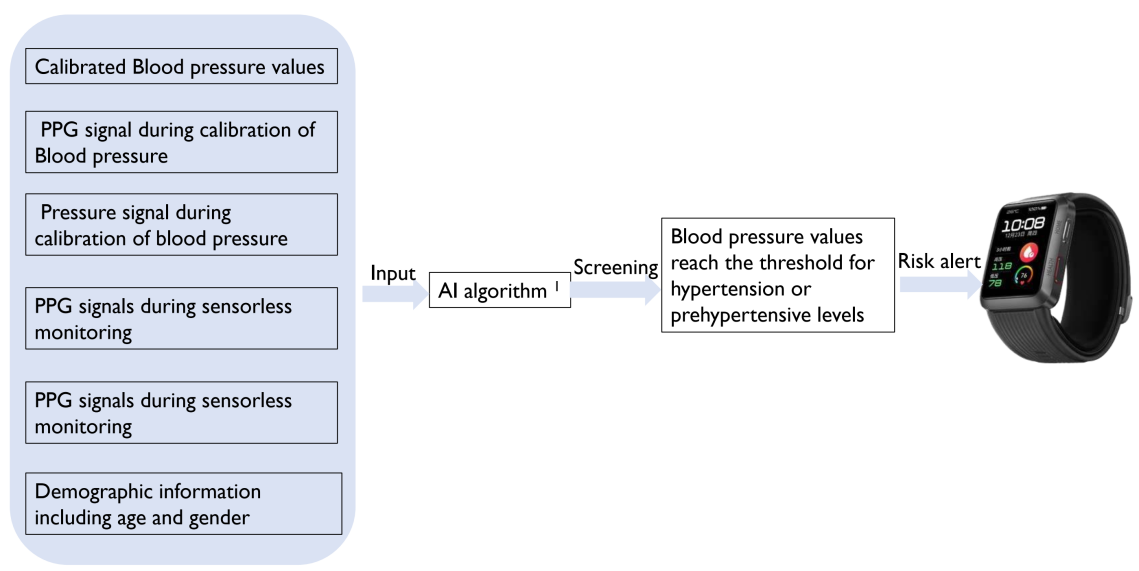


^1^Xiao-yu Fu and Zheng-long Huang (2022). The device, and method for blood pressure measurement (CN115530785A). Huawei Technologies Co., Ltd.

1. The definition of BP output frequency and measurement modes.

| Device function | | Definition |
| --- | --- | --- |
| BP output frequency | Continuous | Outputs BP readings every ≤30 seconds |
|  | Intermittent | Outputs BP readings every >30 seconds (usually 30-60 min), or upon user' s own will and initiation. |
| Measurement   mode | Automated | Measures BP for days, weeks, or months without requiring any action by the user for each measurement via a wearable device |
|  | Manual | Measures BP in a single device position via user activation (e.g., user holds a smartphone, smartwatch, or portable device at heart level while still to take a measurement ). |

Supplemental figure 1. Mann-Kendall test for the mutation point of blood pressure monitoring rate in participants received risk alarms.


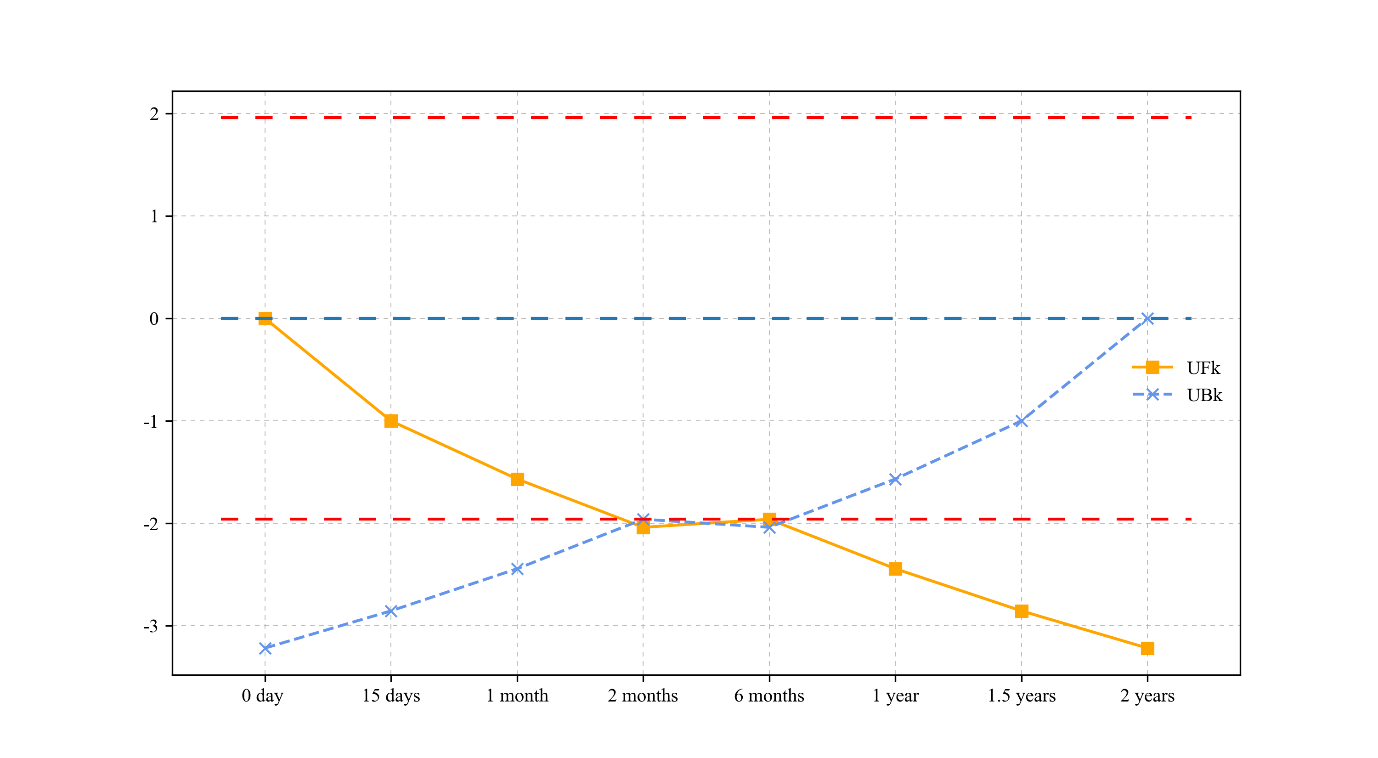


Supplemental figure 2. Mann-Kendall test for the mutation point of blood pressure monitoring rate in hypertensive.


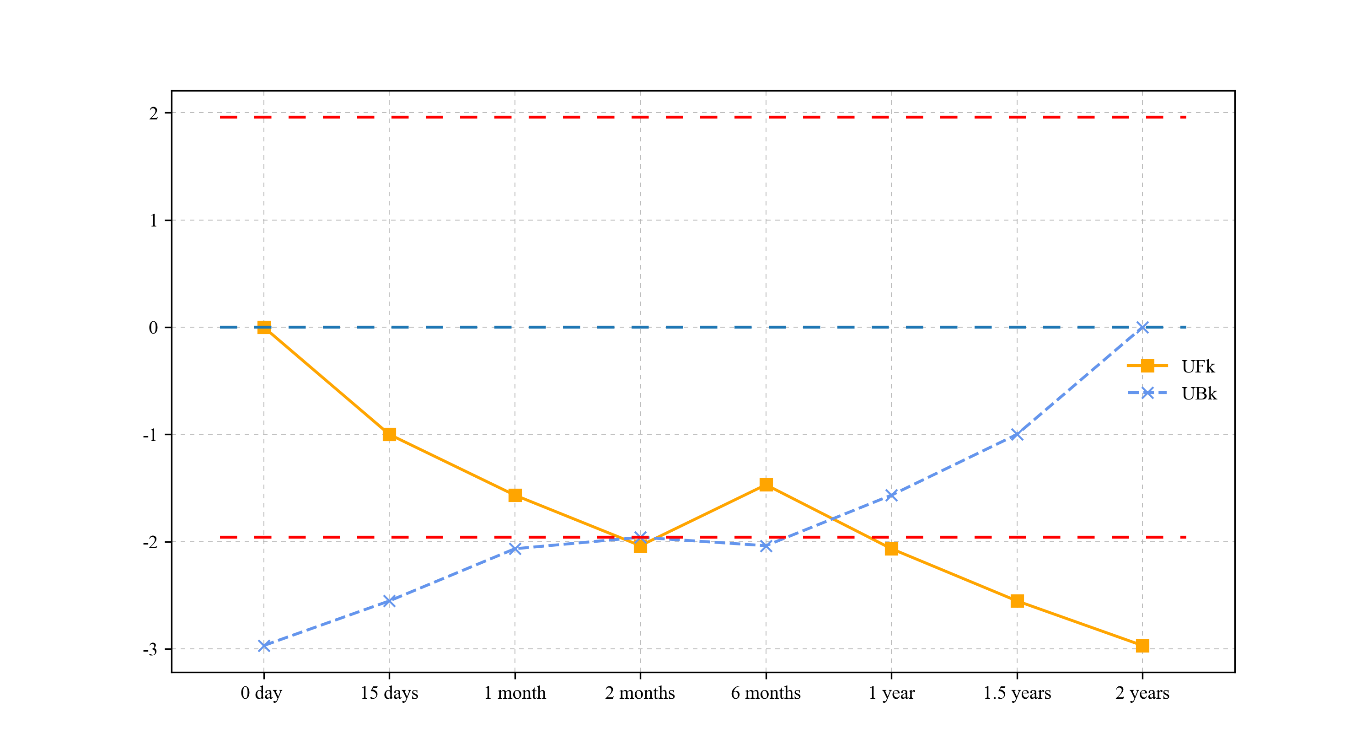

Supplement: ztaf088_Supplementary_Data [file ztaf088_supplementary_data.docx]
